# Cortical activation and functional connectivity during dual-task walking under different cognitive loads: an fNIRS study

**DOI:** 10.3389/fnhum.2026.1906359

**Published:** 2026-09-17

**Authors:** Xinyu Xu, Shurui Li, Sijie Tan, Mei Wang, Qingwen Li, Zhen Guo

**Affiliations:** 1 Tianjin Key Laboratory of Sports Health Integration and Health Promotion, Tianjin Sport University, Tianjin, China; 2 School of Sport Science, Beijing Sport University, Beijing, China; 3 School of Sports Health, Tianjin University of Sport, Tianjin, China

**Keywords:** brain activation, cognitive load, cognitive-motor dual-task, functional connectivity, functional near-infrared spectroscopy, n-back task

## Abstract

**Background:**

Cognitive-motor dual-task processing is common in daily life, but load-related changes in cortical activation and functional connectivity during walking remain incompletely characterized. This study examined neural and behavioral responses to different cognitive demands using functional near-infrared spectroscopy (fNIRS).

**Methods:**

Thirty healthy undergraduate students completed single-task walking (ST), 1-back dual-task walking (DT1), and 2-back dual-task walking (DT2). Hemodynamic signals from prefrontal, motor, and parietal regions were recorded using portable fNIRS. Gait parameters, cognitive accuracy, dual-task cost, and the region-of-interest (ROI) based functional connectivity were analyzed.

**Results:**

Cortical activation showed task-dependent regional changes. Functional connectivity was higher during DT1 and DT2 than during ST (*p* < 0.001), while cognitive and gait performance worsened as task demands increased (*p* < 0.05). Activation-workload associations also differed across conditions.

**Conclusion:**

In healthy young adults, increasing cognitive demands during dual-task walking were associated with distinct changes in cortical activation, interregional connectivity, and behavioral performance. These findings characterize load-related patterns in cortical activation and functional connectivity during dual-task walking.

## 1 Introduction

In everyday life, people often perform cognitive activities while walking, communicating, using electronic devices, or processing environmental information, making cognitive-motor dual-tasking a common behavioral phenomenon (Al-Yahya et al., 2011). A cognitive-motor dual-task refers to the simultaneous performance of cognitive and motor tasks (Watanabe and Funahashi, 2018). Experimental studies show that dual-tasking requires dynamic allocation of limited cognitive resources, and increased cognitive load may impair cognitive and motor performance due to resource competition (Schaefer, 2014). fNIRS studies indicate that higher cognitive load during cognitive-motor dual-task walking is associated with reduced cognitive performance, slower gait speed, and increased prefrontal cortex (PFC) activation (Rahman et al., 2021), along with coordinated changes in motor-related brain regions, suggesting involvement of distributed brain networks (Ou et al., 2024). Therefore, cognitive-motor dual-task processing involves coordinated interactions across multiple brain regions rather than a single region. The PFC, particularly the dorsolateral prefrontal cortex (DLPFC), plays a key role in cognitive control and task regulation (Yang et al., 2013; Chen et al., 2026), while the primary motor cortex (M1) and posterior parietal cortex (PPC) contribute to motor control and sensory integration during dual-task performance (Lim et al., 2022). Overall, examining brain activation and network-level changes under different cognitive loads is essential for understanding its neural mechanisms.

fNIRS, as a non-invasive brain functional imaging technique, can reflect cortical neural activity by monitoring changes in the concentration of oxygenated hemoglobin (HbO). Compared to functional magnetic resonance imaging, fNIRS is relatively robust to motion artifacts and highly portable, making it particularly suitable for investigating brain function during cognitive–motor dual-task walking (Gateau et al., 2018). Therefore, fNIRS has been widely applied in studies on walking, balance control, and dual-task scenarios.

Although previous fNIRS studies have shown that cognitive-motor dual-tasking alters cortical activation, recent work has increasingly incorporated network-level measures and brain-behavior relationships (Ou et al., 2024; Ding et al., 2024). However, relatively few studies have specifically compared parametrically defined working-memory loads during walking while concurrently examining regional activation, interregional functional connectivity, behavioral dual-task costs, and subjective workload within the same participants. Building on these studies, the present work uses a within-subject narrow-base walking paradigm with 1-back and 2-back loads and integrates ROI- and channel-level activation, ROI-to-ROI functional connectivity, gait and cognitive dual-task costs, and NASA-TLX workload. The contribution is therefore an integrated characterization of load-related neural and behavioral changes.

We hypothesized that increasing cognitive demand from ST to DT1 and DT2 would be accompanied by changes in cortical activation and functional connectivity together with poorer cognitive and gait performance. We further explored whether associations between cortical activation and subjective workload differed across task conditions. Accordingly, the study aimed to characterize how regional activation, interregional connectivity, behavioral performance, and perceived workload vary across two cognitive-load levels during dual-task walking in healthy young adults.

## 2 Materials and methods

### 2.1 Participants

Thirty healthy undergraduate students from a university in Tianjin were recruited, including 15 males and 15 females. Their mean age was 21.13 ± 2.42 years old, the mean height was 170.88 ± 7.81 cm, the mean body mass was 65.88 ± 12.94 kg, and the mean score of Montreal Cognitive Assessment (MoCA) was 28.70 ± 0.43 points.

#### 2.1.1 Inclusion criteria

Non-athlete undergraduate students aged 18–24 years; normal physical function enabling participation in regular exercise; no severe visual or auditory impairments; no cognitive impairments (MoCA score≥26) and capable of understanding and cooperating to complete all experimental tasks.

#### 2.1.2 Exclusion criteria

Presence of severe cardiac, pulmonary, or skeletal muscle diseases; presence of neurological disorders significantly affecting motor function; presence of psychiatric conditions such as depression or use of psychotropic medications. The required sample size was calculated using G*Power software (Kang, 2021) (version 3.1, University of Kiel, Kiel, Germany). The statistical test employed in this study was a one-way repeated-measures analysis of variance (repeated-measures ANOVA). The input parameters were set as follows: effect size = 0.25, α error probability = 0.05, power (1-β error probability) = 0.8, and number of groups = 3. The calculation indicated that 28 participants were needed. Considering potential participant dropout and other uncontrollable factors during the experiment, 30 participants were ultimately recruited to ensure an adequate effective sample size. Prior to the fNIRS experiment, all participants provided written informed consent in accordance with the Declaration of Helsinki prior to participation in the experiment. This study was approved by the Ethics Committee of Tianjin Sport University (Ethics Approval Number: TJUS2023-037; approval date: January 7, 2024).

### 2.2 Behavioral tasks

This study employed a repeated-measures experimental design comprising five task conditions: single-task walking (ST), cognitive-only 1-back (CO1), cognitive-only 2-back (CO2), 1-back dual-task walking (DT1), and 2-back dual-task walking (DT2). In the ST condition, participants performed the walking task only, without a concurrent cognitive task. In the CO1 and CO2 conditions, participants performed the corresponding n-back cognitive task while remaining stationary. In the DT1 and DT2 conditions, participants performed the 1-back and 2-back tasks, respectively, while simultaneously walking on the treadmill. The formal task duration was 3 min for each of the five experimental conditions. In the ST condition, participants performed the walking task only, without a concurrent cognitive task. In the CO1 and CO2 conditions, participants performed the corresponding n-back cognitive task while remaining stationary. Under dual-task conditions, participants walked on a treadmill at a speed of 4.0 km/h along a narrow pathway (Wenhua et al., 2022) (path width set to 50% of the inter-anterior superior iliac spine distance) (Mazaheri et al., 2017) while simultaneously performing an auditory n-back working memory task to simulate real-world walking constraints. All experimental procedures were completed over three separate experimental days, with a 24-h interval between consecutive sessions. The three experimental days consisted of a single-task walking session, a 1-back-related session, and a 2-back-related session. The order of the three experimental days was randomized to minimize potential order and learning effects. The ST condition was completed during the single-task walking session; CO1 and DT1 were completed during the 1-back session; and CO2 and DT2 were completed during the 2-back session. The n-back task is a widely used cognitive paradigm for assessing working memory and executive control functions. Its typical procedure involves participants determining whether the current stimulus matches the one presented n trials earlier in a continuous sequence, requiring continuous updating, monitoring, and comparison of memory content (Owen et al., 2005). By parametrically manipulating the value of n, this task systematically varies cognitive load levels, enabling researchers to investigate the modulation of neural activity in response to load changes. Meta-analyses indicate that the n-back task consistently activates brain regions such as the left/right dorsolateral prefrontal cortex (LDLPFC/RDLPFC), PPC, and fronto-parietal network, supporting its reliability as a paradigm for measuring working memory processing mechanisms. In this study, cognitive load was modulated by adjusting the n value (*n* = 1 or 2), requiring participants to continuously update, monitor, and compare auditory stimuli. Each task lasted approximately 2 min, with short rest periods between tasks. After completion of each experimental condition, participants completed the NASA Task Load Index (NASA-TLX) to assess subjective workload. The ST condition served as the single-task baseline for walking performance, whereas CO1 and CO2 served as the cognitive single-task baselines for the corresponding 1-back and 2-back dual-task conditions, respectively.

#### 2.2.1 Cognitive tasks

The cognitive tasks (Linqiong et al., 2022) were programmed, presented, and recorded using E-Prime 3.0 software (Psychology Software Tools, Pittsburgh, USA). The stimuli consisted of a pseudo-random sequence of digits (0–9) delivered auditorily via headphones. Each digit was presented for 500 ms, followed by a 1-s blank interval. Participants were required to compare the currently presented digit with the digit presented n trials earlier (where *n* = 1 or 2). If the digits matched, they responded by pressing a handheld responder with minimal movement; if they did not match, no response was required. Each cognitive load condition comprised three blocks. For both the 1-back and 2-back tasks, each block contained 31 stimuli, with approximately one-third requiring a response. A 20-s rest period was provided between blocks to minimize fatigue-related interference with experimental results. In the CO1 and CO2 conditions, participants performed the corresponding cognitive task while remaining stationary. Cognitive performance was quantified using n-back response accuracy and was subsequently used as the single-task cognitive baseline for calculation of cognitive dual-task cost.

Prior to the formal experiment, the experimenter provided detailed instructions on the experimental procedure and conducted practice sessions to ensure participants understood the task requirements and minimized head movements during the tasks. During the experiment, gait analysis equipment and the portable fNIRS system synchronized data collection. All experiments were conducted in a quiet laboratory environment with controlled lighting and temperature.

#### 2.2.2 Walking tasks

To better approximate the spatial constraints and environmental obstacles that individuals may encounter during walking in real-world scenarios, this study employed a narrow-path walking paradigm to enhance the ecological validity of the experimental task (Mazaheri et al., 2017). A narrow walking path was marked on the treadmill with graphics, and the width of the path was standardized to 50% of the distance between the subjects’ anterior superior iliac spines (Mazaheri et al., 2017). The walking speed was set at 4.0 km/h, which corresponds to the leisure walking speed of Chinese adults (Wenhua et al., 2022). Upon receiving the “experiment start” command from the experimenter, the subjects walked along the narrow path on a PRECOR-C932I programmable treadmill (Precor, USA) at a speed of 4.0 km/h for 3 min, while wearing an emergency stop safety clip throughout the experiment to prevent falls.

The OptoJump Next motion testing system (Microgate, Italy) was positioned on both sides of the treadmill. It utilized infrared sensing devices and video recording equipment to collect and save various spatiotemporal gait parameters of the subjects during the experiment. The walking performance metrics included stride length, step length, cadence, stance phase, and swing phase.

#### 2.2.3 Dual tasks

During DT1 and DT2, participants performed the auditory 1-back and 2-back tasks, respectively, while walking along the narrow treadmill path at 4.0 km/h. The cognitive-task procedure and walking protocol were identical to those used in the corresponding cognitive-only conditions (CO1/CO2) and the single-task walking condition (ST), respectively. Participants were instructed to attend to both tasks without prioritizing either one. Dual-task cost (DTC) was calculated as the relative change in performance between the dual-task and corresponding single-task conditions (Linqiong et al., 2022). For walking performance, ST served as the baseline for both DT1 and DT2. Depending on whether a higher value indicated better or poorer performance, walking DTC was calculated as: DTC = (DT - ST) / ST × 100% or DTC = (ST - DT) / ST × 100% (Feng et al., 2025), where ST and DT represent the corresponding gait parameters during single-task walking and dual-task walking, respectively. For cognitive performance, n-back accuracy during CO1 and CO2 served as the baselines for DT1 and DT2, respectively. Cognitive DTC was calculated as: Cognitive DTC = (CO-DT) / CO × 100%, where CO and DT represent n-back accuracy during the corresponding cognitive-only and dual-task conditions, respectively. A larger positive cognitive DTC indicated a greater reduction in cognitive performance during dual-task walking.

### 2.3 fNIRS

#### 2.3.1 fNIRS acquisition

In this study, a portable fNIRS system, the NirSmartII-100Pro (Danyang Huichuang Medical Equipment Co., Ltd., China), was employed to measure the concentration changes of HbO and deoxygenated hemoglobin (HbR) in the cerebral cortex of subjects during different tasks. The instrument utilized a near-infrared light source and detector array to form multi-channel measurements, covering brain regions associated with working memory and motor control, including the PFC, M1, and PPC. This device captured HbO concentration changes using two wavelengths of near-infrared light—760 nm and 850 nm—at a sampling frequency of 11 Hz. The imaging cap was designed based on the international 10/20 standard electrode placement system, consisting of 24 light source emitters and 24 light detectors, forming a total of 69 channels. During the ROI delineation process, channels that could not be accurately mapped to specific brain regions were excluded, resulting in approximately 65 channels included in the final analysis. The emitters and detectors were spaced 30 mm apart to form detection channels (Figures 1, 2). These channels covered multiple ROIs of interest, including the left and right prefrontal cortices (LPFC/RPFC), dorsolateral prefrontal cortices (LDLPFC/RDLPFC), motor cortices (LM1/RM1), somatosensory cortices (LS1/RS1), and posterior parietal cortices (LPPC/RPPC) (Table 1). The selection of these ROIs was based on Brodmann areas (BA) and anatomical references related to motor and cognitive processes.

**Figure 1:**
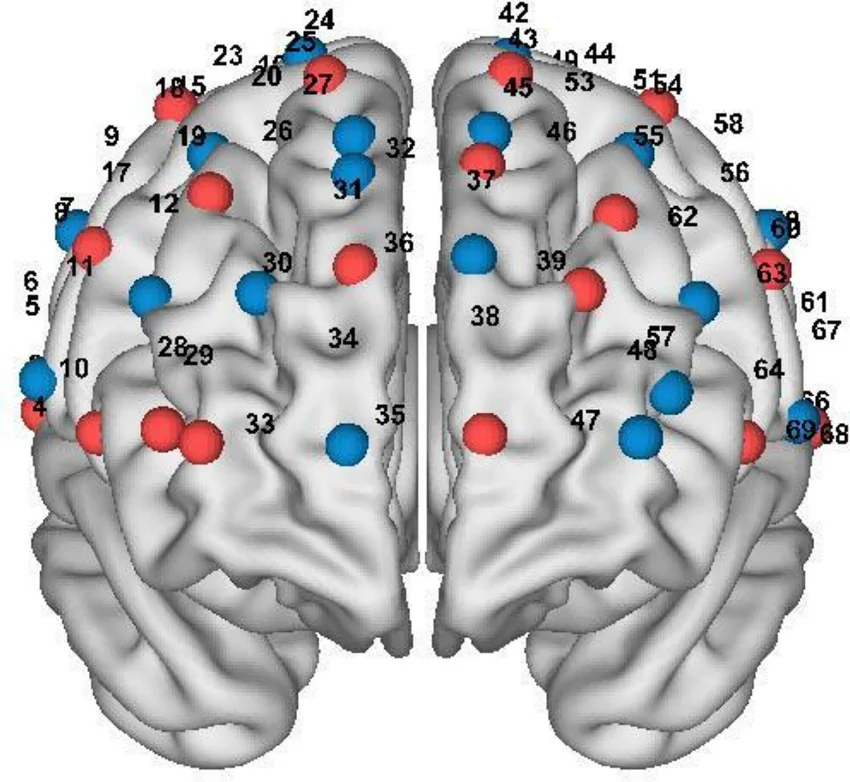
Schematic of fNIRS channel arrangement.

**Figure 2:**
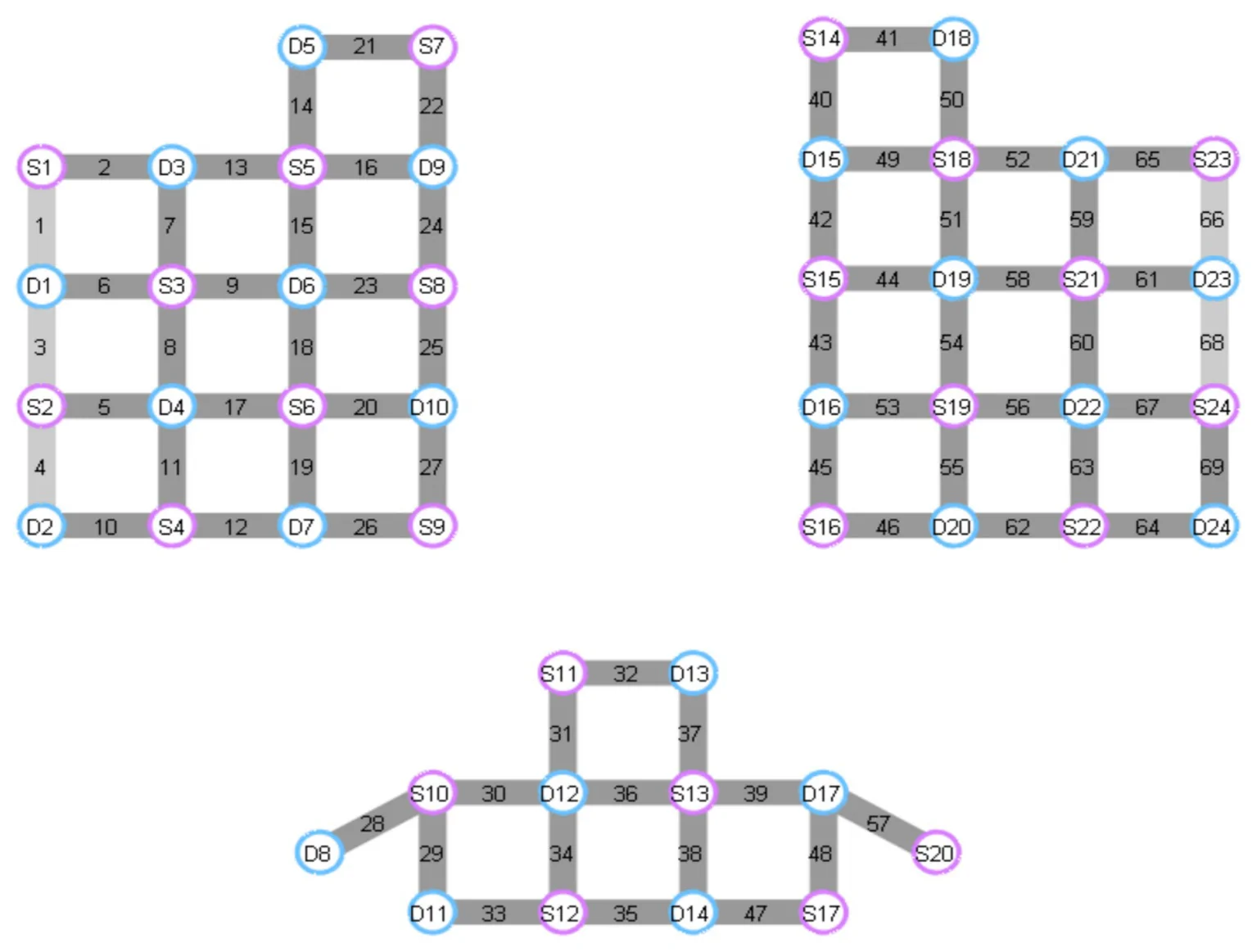
Template in the fNIRS collection.

**Table 1:** The MNI coordinates and ROI corresponding to the measurement channels.

Channel number	S-D	X	Y	Z	ROI
CH2	S1-D3	61.3333	−58.3333	36.3333	RPPC
CH5	S2-D4	67.3333	−4.6667	33	RS1
CH6	S3-D1	69	−33.6667	36.6667	RPPC
CH7	S3-D3	62.3333	−42.3333	49.6667	RPPC
CH8	S3-D4	63	−17	47.6667	RS1
CH9	S3-D6	54.3333	−27.6667	59.6667	RS1
CH10	S4-D2	60	24.6667	22.6667	RDLPFC
CH11	S4-D4	56.6667	11	42	RM1
CH12	S4-D7	44.6667	26.6667	48.6667	RDLPFC
CH13	S5-D3	45.6667	−54.6667	60	RPPC
CH14	S5-D5	34.6667	−64.6667	62.6667	RPPC
CH16	S5-D9	25.3333	−52.6667	74	RPPC
CH17	S6-D4	51.6667	−0.33333	55.6667	RDLPFC
CH18	S6-D6	43.3333	−15.6667	67.6667	RM1
CH19	S6-D7	39.6667	14	61	RDLPFC
CH20	S6-D10	28.3333	−0.33333	69.6667	RDLPFC
CH21	S7-D5	21.6667	−75.6667	60.6667	RPPC
CH22	S7-D9	15.6667	−63.3333	72	RPPC
CH23	S8-D6	33.3333	−26.6667	74	RM1
CH24	S8-D9	18	−39	79.6667	RS1
CH25	S8-D10	20.3333	−12.6667	76.3333	RM1
CH26	S9-D7	23.6667	26.3333	61.6667	RDLPFC
CH27	S9-D10	16	13	71	RDLPFC
CH28	S10-D8	44.3333	50.3333	25.6667	RDLPFC
CH29	S10-D11	38.3333	57.3333	24.3333	RDLPFC
CH30	S10-D12	24.6667	53.6667	40.6667	RDLPFC
CH31	S11-D12	13.3333	46.6667	52.3333	RPFC
CH32	S11-D13	1.6667	34.3333	57	LPFC
CH33	S12-D11	28.3333	68.6667	11.6667	RDLPFC
CH34	S12-D12	15.3333	66.3333	28.6667	RDLPFC
CH35	S12-D14	4.3333	70.3333	13.6667	RPFC
CH36	S13-D12	1.3333	55	40.6667	LPFC
CH37	S13-D13	−10.3333	45.6667	52.3333	LPFC
CH38	S13-D14	−12.3333	64.3333	30.6667	LDLPFC
CH39	S13-D17	−19.6667	52.6667	41.6667	LDLPFC
CH40	S14-D15	−14.6667	−63.6667	71.3333	LPPC
CH41	S14-D18	−21	−76	59.6667	LPPC
CH42	S15-D15	−15.6667	−39.3333	79.3333	LS1
CH43	S15-D16	−17.3333	−12.6667	77.6667	LM1
CH44	S15-D19	−29.6667	−24.3333	74	LM1
CH45	S16-D16	−15	13.3333	70.6667	LDLPFC
CH46	S16-D20	−23.6667	26.6667	61	LDLPFC
CH47	S17-D14	−27.6667	67	12.6667	LDLPFC
CH48	S17-D17	−36.3333	55.3333	25.6667	LDLPFC
CH49	S18-D15	−24.3333	−52.3333	74	LPPC
CH50	S18-D18	−34.6667	−64.3333	62.6667	LPPC
CH51	S18-D19	−38.3333	−38.6667	69.6667	LS1
CH52	S18-D21	−46.6667	−56.3333	57.6667	LPPC
CH53	S19-D16	−28	1	69	LDLPFC
CH54	S19-D19	−41.3333	−14.6667	67.6667	LM1
CH55	S19-D20	−38.6667	13.6667	61	LDLPFC
CH56	S19-D22	−51.6667	−2.6667	54.6667	LM1
CH57	S20-D17	−40.6667	49.6667	27.6667	LDLPFC
CH58	S21-D19	−52	−27.3333	61.3333	LS1
CH59	S21-D21	−62	−44.6667	46.6667	LPPC
CH60	S21-D22	−63	−19.3333	44.3333	LPPC
CH61	S21-D23	−68	−34.6667	32.3333	LPPC
CH62	S22-D20	−44.3333	25.3333	46.3333	LDLPFC
CH63	S22-D22	−57.3333	8.3333	38.3333	LM1
CH64	S22-D24	−58.6667	23	21	LDLPFC
CH65	S23-D21	−60.3333	−61.6667	31.3333	LPPC
CH67	S24-D22	−67	−7.3333	27.3333	LS1
CH69	S24-D24	−63.6667	6.3333	11	LPFC

To ensure standardized anatomical localization of the fNIRS measurement channels, anatomical mapping was performed using standard template registration. The spatial coordinates of the optodes and measurement channels were transformed into Montreal Neurological Institute (MNI) coordinates and registered to the MNI standard brain template using NirSpark software (Danyang Huichuang Medical Equipment Co., Ltd., China) (Li et al., 2020, 2023). Based on the registered MNI coordinates and the anatomical information provided by NirSpark, each measurement channel was assigned to the corresponding cortical region. The resulting anatomical locations were further referenced to Brodmann areas and cortical anatomical landmarks to define the ROIs.

#### 2.3.2 fNIRS analysis

The selected ROIs included the LM1 (channels 43, 44, 54, 56, 63), RM1 (channels 11, 18, 23, 25), LS1 (channels 42, 51, 58, 67), RS1 (channels 5, 8, 9, 15, 24), LPPC (channels 40, 41, 49, 50, 52, 59, 60, 61, 65), RPPC (channels 2, 6, 7, 13, 14, 16, 21, 22), LPFC (channels 32, 36, 37, 69), RPFC (channels 31, 35), LDLPFC (channels 38, 39, 45, 46, 47, 48, 53, 55, 57, 62, 64), and RDLPFC, (channels 10, 12, 17, 19, 20, 26, 27, 28, 29, 30, 33, 34). To ensure the specificity of the analyzed regions, the ROIs for the LPFC and RPFC were defined by excluding the areas corresponding to the LDLPFC and RDLPFC. The selection of ROIs was based on their functional roles in motor control, somatosensory processing, and executive control. To avoid overlap with the LDLPFC and RDLPFC, the ROIs for the LPFC and RPFC were defined excluding LDLPFC and RDLPFC regions. Before each formal recording session, the signal quality of all channels was thoroughly evaluated using a data quality analysis tool. To ensure the reliability of subsequent analyses and spatial consistency of the data, any channels that failed to meet the quality standards were excluded from further analysis of brain activation and functional connectivity. The preprocessing methods for fNIRS data included bad segment removal, motion artifact correction, and filtering. We used the preprocessing module of NirSpark software (Danyang Huichuang Medical Equipment Co., Ltd., China) to preprocess the collected data (Li et al., 2020, 2023). During the data preprocessing stage, signal segments with obvious abnormal fluctuations, which may result from excessive subject movement or slight probe displacement, were first identified and removed. Abnormal detection employed a standard deviation threshold of 6.0 and an amplitude threshold of 0.5 to ensure signal stability. Subsequently, motion artifacts were corrected using a comprehensive method based on moving standard deviation and cubic spline interpolation. To reduce interference from physiological rhythmic fluctuations, a bandpass filter (0.01–0.2 Hz) was applied to remove high-frequency and low-frequency components such as heart rate, respiration, and Mayer waves. Finally, the Modified Beer–Lambert Law (MBLL) was used to convert optical intensity signals into relative concentration changes of HbO and HbR, with the differential pathlength factor (DPF) set to 6.6. For data segmentation and preprocessing in NirSpark, a broader signal range of 10–210 s was retained to include relatively stable signal periods surrounding the formal task and to reduce the influence of transient hemodynamic changes occurring around task onset and termination. Importantly, this 10–210 s range was used only for signal retention and preprocessing and did not represent the final analysis window. For all subsequent statistical analyses, the data were segmented according to the experimental event markers, and only the 180 s signal corresponding to the formal task period was included. Signals from the pre-task and post-task resting periods were not included in either cortical activation or FC analyses. No explicit short-separation regression or additional systemic-signal regression was performed. Therefore, although the 0.01–0.20 Hz band-pass filter was used to attenuate major physiological fluctuations, residual extracerebral or systemic physiological contributions cannot be completely excluded. For cortical activation analysis, the complete 180 s HbO signal corresponding to each formal task condition was extracted. The mean change in HbO concentration over the task period was calculated for each measurement channel and ROI to characterize task-related cortical activation. FC analysis was performed using the Batch Analysis Tool-FC and network modules of NirSpark software (Danyang Huichuang Medical Equipment Co., Ltd., China). For each task condition (ST, DT1, and DT2), the average time series of all channels within each ROI was first calculated, the complete 180 s HbO time series corresponding to the formal task period was extracted. Subsequently, Pearson correlation analysis was used to compute the correlation coefficients between the time series of each pair of channels, generating a 10 × 10 functional connectivity matrix for each subject. To improve data normality, the correlation coefficients underwent Fisher’s r-to-z transformation. Based on this, the average z-scores between each pair of ROIs were calculated, and the global mean FC value was further derived to reflect the overall level of global functional connectivity. All statistical results were subjected to false discovery rate (FDR) correction (q < 0.05) to control for multiple comparison errors.

### 2.4 Statistical analysis

Statistical analyses were conducted using SPSS 27.0 software. Initially, the Shapiro–Wilk test was employed to assess data normality, and Levene’s test was used to evaluate homogeneity of variance. For repeated-measures data, Mauchly’s test of sphericity was further applied to assess the sphericity assumption; when this assumption was violated, the Greenhouse–Geisser correction was applied. For normally distributed variables, including gait parameters such as stride length, step length, cadence, stance phase, and swing phase, cognitive performance measured by n-back accuracy, and fNIRS metrics including ROI activation, channel activation, and functional connectivity, repeated-measures analysis of variance (ANOVA) was conducted to examine the main effect of task condition (ST, DT1, and DT2). When a significant main effect was identified, *post hoc* pairwise comparisons were performed with Bonferroni correction. For variables that did not follow a normal distribution, the Friedman test was used for overall comparisons, followed by Wilcoxon signed-rank tests for pairwise comparisons when the overall effect was significant. To account for multiple testing, the Benjamini-Hochberg false discovery rate (FDR) correction was applied separately to families of comparisons involving multiple ROIs, multiple channels, multiple ROI-to-ROI functional connectivity pairs, multiple NASA-TLX workload dimensions, and multiple brain–workload correlation analyses. An FDR-adjusted significance threshold of q < 0.05 was used. For functional connectivity analysis, Pearson correlation coefficients (r) between ROIs were first calculated for each participant under each task condition and subsequently transformed using Fisher’s z transformation to improve normality. The resulting z values were used for group-level statistical analyses, including repeated-measures ANOVA or the Friedman test, as appropriate, as well as subsequent pairwise comparisons. For visualization and descriptive presentation of functional connectivity results, the original Pearson correlation coefficients (r) were reported. For comparisons involving two paired conditions, paired-samples t-tests were used for normally distributed data, whereas Wilcoxon signed-rank tests were applied when the normality assumption was not satisfied. Effect sizes were reported where appropriate, including partial η^2^ for repeated-measures ANOVA and Cohen’s dz. for paired-samples comparisons, together with 95% confidence intervals. Statistical significance was set at *p* < 0.05 unless otherwise specified after multiple-comparison correction.

## 3 Results

### 3.1 fNIRS data

#### 3.1.1 Brain activation

Figures 3A,B and Table 2 illustrate the brain activation patterns in various ROIs on both sides under different task conditions. The results revealed that for the RM1, RPFC, RDLPFC, LM1, LPFC, LDLPFC, and LPPC, repeated-measures ANOVA demonstrated a significant main effect of task (all *p* < 0.05).

**Figure 3:**
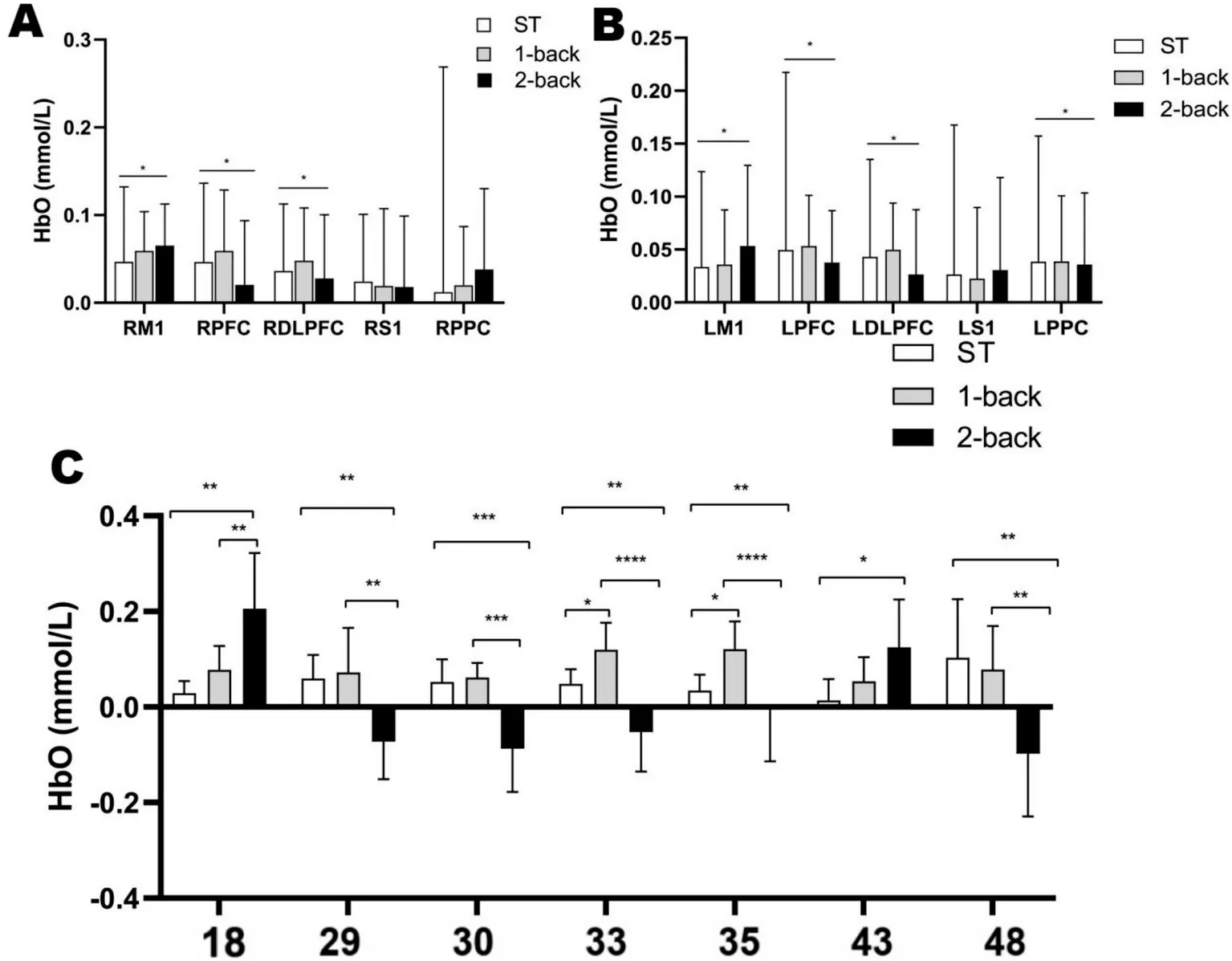
Cortical activation patterns under different task conditions. **(A)** Right hemisphere activation, **(B)** Left hemisphere activation, **(C)** Channel-wise activation. *p < 0.05, **p < 0.01, ***p < 0.001, ****p < 0.0001.

**Table 2:** Main effects of task on cortical activation across predefined ROIs.

ROI	*F*	df	Adjusted *p*	Partial *η*^2^	95% CI for partial *η*^2^
RM1	17.22	2, 58	<0.001	0.373	[0.174, 0.525]
LM1	7.40	2, 58	0.014	0.203	[0.039, 0.368]
RPFC	15.67	2, 58	<0.001	0.351	[0.154, 0.506]
LPFC	11.88	2, 58	<0.001	0.291	[0.101, 0.453]
RDLPFC	15.79	2, 58	<0.001	0.353	[0.156, 0.508]
LDLPFC	15.46	2, 58	<0.001	0.348	[0.151, 0.504]
LPPC	10.00	2, 58	0.002	0.256	[0.075, 0.420]

Furthermore, comparisons of activation levels across the three task conditions (ST, DT1, and DT2) revealed significant task-related differences in Channels 18, 29, 30, 33, 35, 43, and 48 (Figure 3C and Table 3). Specifically, Channel 18 showed significantly higher activation during DT1 than during DT2, while activation during ST was also significantly higher than that during DT2; no significant difference was observed between ST and DT1 (ST: 0.078 ± 0.047; DT1: 0.206 ± 0.111; DT2: 0.013 ± 0.043). Channel 29 showed significantly higher activation during both DT1 and ST than during DT2, whereas no significant difference was observed between ST and DT1 (ST: 0.060 ± 0.047; DT1: 0.072 ± 0.088; DT2: −0.073 ± 0.075). Similarly, Channel 30 showed significantly higher activation during both DT1 and ST than during DT2, with no significant difference between ST and DT1 (ST: 0.052 ± 0.045; DT1: 0.062 ± 0.029; DT2: −0.087 ± 0.086). Channel 33 showed significantly higher activation during DT1 than during both ST and DT2, while activation during ST was also significantly higher than that during DT2 (ST: 0.049 ± 0.029; DT1: 0.120 ± 0.054; DT2: −0.052 ± 0.079). Channel 35 exhibited a similar pattern, with significantly higher activation during DT1 than during both ST and DT2, and significantly higher activation during ST than during DT2 (ST: 0.034 ± 0.032; DT1: 0.121 ± 0.055; DT2: −0.003 ± 0.105). Channel 43 showed significantly higher activation during DT2 than during ST, whereas neither DT1 versus DT2 nor ST versus DT1 reached statistical significance (ST: 0.054 ± 0.048; DT2: 0.125 ± 0.095). Finally, Channel 48 showed significantly higher activation during DT2 than during DT1 and significantly higher activation during ST than during DT2, whereas no significant difference was observed between ST and DT1 (ST: 0.078 ± 0.087; DT1: −0.097 ± 0.125; DT2: 0.029 ± 0.024).

**Table 3:** Pairwise comparisons of channel-level cortical activation across task conditions.

Channel	Contrast	*n*	*t*	df	Adjusted *p*	Cohen’s dz	95% CI for dz
CH18	DT1 vs. DT2	28	3.30	27	0.005	0.62	[0.22, 1.03]
CH18	ST vs. DT1	28	−1.62	27	0.118	−0.31	[−0.69, 0.07]
CH18	ST vs. DT2	28	3.60	27	0.002	0.68	[0.27, 1.09]
CH29	DT1 vs. DT2	30	3.85	29	0.00147	0.70	[0.30, 1.10]
CH29	ST vs. DT1	30	−0.45	29	0.726	−0.08	[−0.44, 0.28]
CH29	ST vs. DT2	30	3.55	29	0.00243	0.65	[0.26, 1.04]
CH30	DT1 vs. DT2	30	4.70	29	0.000118	0.86	[0.44, 1.28]
CH30	ST vs. DT1	30	−0.42	29	0.726	−0.08	[−0.44, 0.28]
CH30	ST vs. DT2	30	4.55	29	0.000188	0.83	[0.42, 1.25]
CH33	DT1 vs. DT2	29	5.00	28	0.000039	0.93	[0.49, 1.36]
CH33	ST vs. DT1	29	−2.56	28	0.01645	−0.47	[−0.86, −0.09]
CH33	ST vs. DT2	29	3.55	28	0.00240	0.66	[0.26, 1.06]
CH35	DT1 vs. DT2	30	4.85	29	0.000063	0.89	[0.46, 1.31]
CH35	ST vs. DT1	30	−2.40	29	0.02349	−0.44	[−0.81, −0.06]
CH35	ST vs. DT2	30	3.35	29	0.00373	0.61	[0.22, 1.00]
CH43	DT1 vs. DT2	27	1.75	26	0.097	0.34	[−0.05, 0.73]
CH43	ST vs. DT1	27	−1.68	26	0.105	−0.32	[−0.71, 0.06]
CH43	ST vs. DT2	27	−2.85	26	0.011	−0.55	[−0.95, −0.14]
CH48	DT1 vs. DT2	30	−3.00	29	0.00785	−0.55	[−0.93, −0.16]
CH48	ST vs. DT1	30	0.42	29	0.726	0.08	[−0.28, 0.44]
CH48	ST vs. DT2	30	3.30	29	0.00359	0.60	[0.21, 0.99]

Adjusted *p* values are reported as provided. Cohen’s dz represents the paired-samples effect size; CI, confidence interval; ST, single-task walking; DT1, 1-back dual-task walking; DT2, 2-back dual-task walking.

#### 3.1.2 Functional connectivity

The connection strength between channel groups is depicted in Figure 4 and Table 4. The average inter-channel connection strength for the ST was 0.32 ± 0.14 (Figure 4A), for the DT1 it was 0.41 ± 0.14 (Figure 4B), and for the DT2 it was 0.44 ± 0.14 (Figure 4C). Significant differences were observed in the FC values across the three task conditions (*p* < 0.001). *Post hoc* comparisons revealed that the FC under the DT1 was significantly higher than that under the ST (*p* = 0.001), and the FC under the DT2 was also significantly higher than that under the ST condition (*p* < 0.001; Figure 4D).

**Figure 4:**
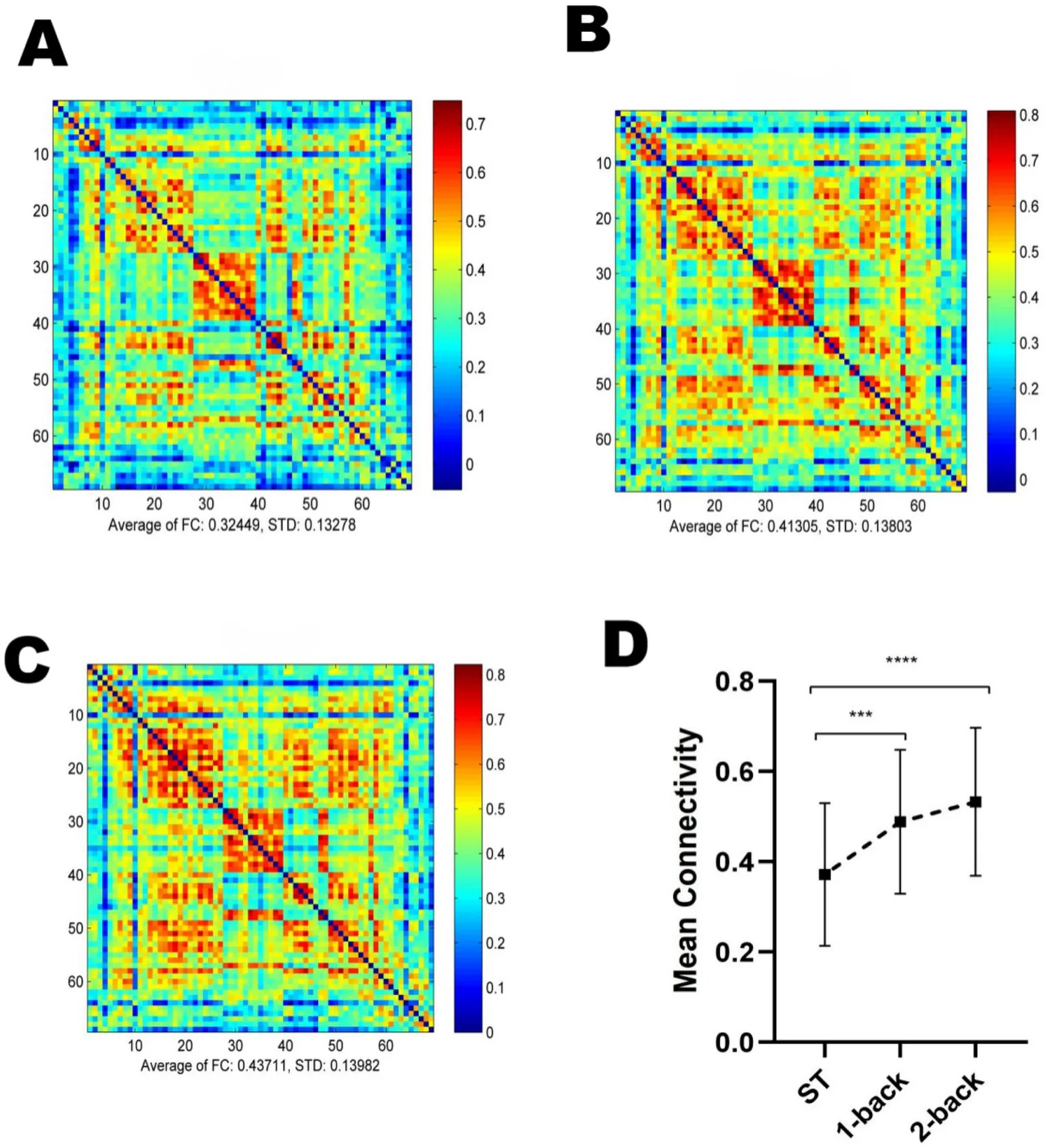
Results of FC of channel-to-channel. **(A)** ST, **(B)** DT1, **(C)** DT2, **(D)** Mean connectivity strength of 65 channels among three groups. *p < 0.05, **p < 0.01, ***p < 0.001, ****p < 0.0001.

**Table 4:** Pairwise comparisons of functional connectivity across task conditions.

Comparison	*n*	*t*	df	Adjusted *p*	Cohen’s dz	95% CI for dz
ST vs. DT1	30	−4.28	29	0.000558	−0.78	[−1.19, −0.37]
ST vs. DT2	30	−6.05	29	0.00000414	−1.10	[−1.56, −0.64]
DT1 vs. DT2	30	−1.90	29	0.202	−0.35	[−0.71, 0.02]

Figure 5 and Table 5 presents the results of ROI-to-ROI FC among the three groups. Between the RPFC and the RPPC, the connection strengths under the DT2 (0.42 ± 0.28) and DT1 (0.35 ± 0.19) conditions were significantly higher than those under the ST (0.27 ± 0.20, *p* = 0.016; *p* = 0.004), while no significant difference was observed between the DT2 and DT1 (*p* = 0.103). Between the RDLPFC and the RPPC, the connection strengths under the DT2 (0.44 ± 0.24) and DT1 (0.38 ± 0.20) were significantly higher than those under the ST (0.31 ± 0.16, *p* = 0.006; *p* < 0.001), but no significant difference was found between the DT2 and DT1 (*p* = 0.050). Between the RDLPFC and the LDLPFC, the connection strengths under the DT2 (0.61 ± 0.17) and DT1 (0.59 ± 0.21) were significantly higher than those under the ST (0.43 ± 0.19, *p* < 0.001; *p* < 0.001), while no significant difference was observed between the DT2 and DT1 (*p* = 0.511). Between the RM1 and the RS1, the connection strengths under the DT2 (0.72 ± 0.35) and DT1 (0.61 ± 0.30) were significantly higher than those under the ST (0.55 ± 0.26, *p* = 0.025; *p* < 0.001), and the DT2 also exhibited significantly higher connection strength than the DT1 (*p* = 0.007). Between the RM1 and the RPPC, the connection strengths under the DT2 (0.57 ± 0.28) and DT1 (0.52 ± 0.27) were significantly higher than those under the ST (0.42 ± 0.26, *p* = 0.001; *p* < 0.001), while no significant difference was observed between the DT2 and DT1 (*p* = 0.146). Between the LPFC and the RPPC, the connection strengths under the DT2 (0.38 ± 0.23) and DT1 (0.35 ± 0.18) were significantly higher than those under the ST (0.19 ± 0.16, *p* < 0.001; *p* < 0.001), but no significant difference was found between the DT2 and DT1 (*p* = 0.492). Between the LPFC and the LPPC, the connection strengths under the DT2 (0.37 ± 0.22) and DT1 (0.37 ± 0.19) were significantly higher than those under the ST (0.20 ± 0.20, *p* = 0.001; *p* < 0.001), while no significant difference was observed between the two conditions (*p* = 0.910). Between the LDLPFC and the LPPC, the connection strengths under the DT2 (0.43 ± 0.23) and DT1 (0.39 ± 0.21) were significantly higher than those under the ST (0.26 ± 0.20, *p* = 0.002; *p* < 0.001), but no significant difference was found between the DT2 and DT1 (*p* = 0.252). Between the LS1 and the RPPC, the connection strengths under the DT2 (0.55 ± 0.27) and DT1 (0.50 ± 0.24) were significantly higher than those under the ST (0.37 ± 0.22, *p* = 0.003; *p* = 0.001), while no significant difference was observed between the two conditions (*p* = 0.075). Between the LM1 and the RS1, the connection strengths under the DT2 (0.61 ± 0.28) and DT1 (0.52 ± 0.27) were significantly higher than those under the ST (0.43 ± 0.23, *p* = 0.023; *p* < 0.001), and the DT2 also exhibited significantly higher connection strength than the DT1 (*p* = 0.035). Between the LM1 and the RPPC, the connection strengths under the DT2 (0.50 ± 0.25) and DT1 (0.46 ± 0.24) were significantly higher than those under the ST (0.35 ± 0.20, *p* = 0.013, *p* = 0.003), while no significant difference was observed between the DT2 and DT1 (*p* = 0.226). Between the LM1 and the LDLPFC, the connection strengths under the DT2 (0.51 ± 0.25) and DT1 (0.46 ± 0.22) were significantly higher than those under the ST (0.34 ± 0.24, *p* < 0.001, *p* < 0.001), but no significant difference was observed between the two conditions (*p* = 0.106).

**Figure 5:**
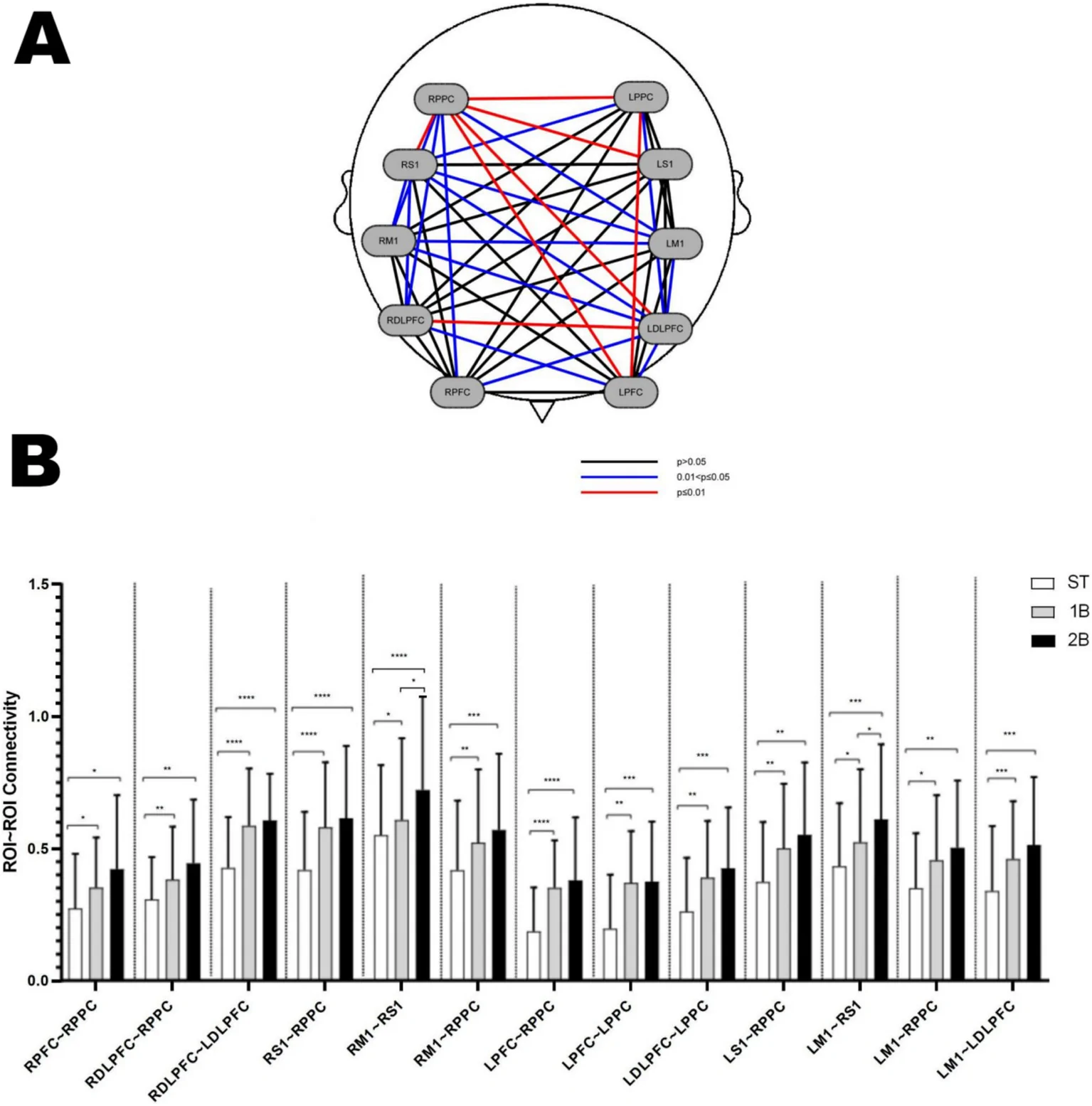
Results of FC of ROI-to-ROI. **(A)** FC of ROI-to-ROI. **(B)** ROI-to-ROI functional connectivity across different task conditions. ST indicates single-task walking; 1B indicates walking under the 1-back task condition; 2B indicates walking under the 2-back task condition. *p < 0.05, **p < 0.01, ***p < 0.001, ****p < 0.0001.

**Table 5:** Pairwise comparisons of ROI-to-ROI functional connectivity among ST, DT1, and DT2.

ROI connection	Comparison	*t*	df	Adjusted *p*	Cohen’s dz	Approx. 95% CI
RPFC–RPPC	DT2 vs. ST	2.75	29	0.016	0.50	[0.13, 0.87]
RPFC–RPPC	DT1 vs. ST	3.38	29	0.004	0.62	[0.24, 0.99]
RPFC–RPPC	DT2 vs. DT1	1.80	29	0.103	0.33	[−0.05, 0.70]
RDLPFC–RPPC	DT2 vs. ST	3.20	29	0.006	0.58	[0.21, 0.96]
RDLPFC–RPPC	DT1 vs. ST	5.05	29	<0.001	0.92	[0.55, 1.29]
RDLPFC–RPPC	DT2 vs. DT1	2.18	29	0.050	0.40	[0.02, 0.77]
RDLPFC–LDLPFC	DT2 vs. ST	4.80	29	<0.001	0.88	[0.50, 1.25]
RDLPFC–LDLPFC	DT1 vs. ST	4.65	29	<0.001	0.85	[0.48, 1.22]
RDLPFC–LDLPFC	DT2 vs. DT1	0.69	29	0.511	0.13	[−0.25, 0.50]
RM1–RS1	DT2 vs. ST	2.52	29	0.025	0.46	[0.09, 0.83]
RM1–RS1	DT1 vs. ST	4.55	29	<0.001	0.83	[0.46, 1.20]
RM1–RS1	DT2 vs. DT1	3.12	29	0.007	0.57	[0.20, 0.94]
RM1–RPPC	DT2 vs. ST	4.04	29	0.001	0.74	[0.36, 1.11]
RM1–RPPC	DT1 vs. ST	4.47	29	<0.001	0.82	[0.44, 1.19]
RM1–RPPC	DT2 vs. DT1	1.58	29	0.146	0.29	[−0.09, 0.66]
LPFC–RPPC	DT2 vs. ST	4.40	29	<0.001	0.80	[0.43, 1.18]
LPFC–RPPC	DT1 vs. ST	4.35	29	<0.001	0.79	[0.42, 1.17]
LPFC–RPPC	DT2 vs. DT1	0.74	29	0.492	0.14	[−0.24, 0.51]
LPFC–LPPC	DT2 vs. ST	4.01	29	0.001	0.73	[0.36, 1.11]
LPFC–LPPC	DT1 vs. ST	4.30	29	<0.001	0.78	[0.41, 1.16]
LPFC–LPPC	DT2 vs. DT1	0.11	29	0.910	0.02	[−0.35, 0.39]
LDLPFC–LPPC	DT2 vs. ST	3.70	29	0.002	0.68	[0.30, 1.05]
LDLPFC–LPPC	DT1 vs. ST	4.25	29	<0.001	0.78	[0.40, 1.15]
LDLPFC–LPPC	DT2 vs. DT1	1.22	29	0.252	0.22	[−0.15, 0.60]
LS1–RPPC	DT2 vs. ST	3.53	29	0.003	0.64	[0.27, 1.02]
LS1–RPPC	DT1 vs. ST	3.99	29	0.001	0.73	[0.35, 1.10]
LS1–RPPC	DT2 vs. DT1	1.97	29	0.075	0.36	[−0.01, 0.73]
LM1–RS1	DT2 vs. ST	2.58	29	0.023	0.47	[0.10, 0.84]
LM1–RS1	DT1 vs. ST	4.22	29	<0.001	0.77	[0.40, 1.14]
LM1–RS1	DT2 vs. DT1	2.36	29	0.035	0.43	[0.06, 0.80]
LM1–RPPC	DT2 vs. ST	2.85	29	0.013	0.52	[0.15, 0.89]
LM1–RPPC	DT1 vs. ST	3.51	29	0.003	0.64	[0.27, 1.01]
LM1–RPPC	DT2 vs. DT1	1.31	29	0.226	0.24	[−0.13, 0.61]
LM1–LDLPFC	DT2 vs. ST	4.18	29	<0.001	0.76	[0.39, 1.14]
LM1–LDLPFC	DT1 vs. ST	4.15	29	<0.001	0.76	[0.38, 1.13]
LM1–LDLPFC	DT2 vs. DT1	1.76	29	0.106	0.32	[−0.05, 0.70]

Overall, as task load gradually increased from ST to DT1 and then to DT2, the functional connectivity strength between most ROIs exhibited an increasing trend, with significantly higher connectivity under the DT2 compared to the ST. This suggests that higher cognitive load enhances the functional connectivity strength between the prefrontal-parietal and sensorimotor networks.

### 3.2 Behavioral data

#### 3.2.1 Walking performance

Compared with ST, DT1 was associated with significantly shorter stride length, *t*(29) = 2.52, adjusted *p* = 0.035, Cohen’s dz. = 0.46, 95% CI [0.08, 0.84], and step length, *t*(29) = 2.67, adjusted *p* = 0.027, dz. = 0.49, 95% CI [0.10, 0.87]. Cadence was significantly higher during DT1 than during ST, *t*(29) = −2.73, adjusted *p* = 0.025, dz. = −0.50, 95%CI [−0.88, −0.11]. In contrast, the differences in stance phase duration, *t*(29) = 1.18, adjusted *p* = 0.328, dz. = 0.22, 95% CI [−0.15, 0.58], and swing phase duration, *t*(29) = 1.52, adjusted *p* = 0.172, dz. = 0.28, 95%CI [−0.09, 0.64], were not statistically significant.

Compared with ST, DT2 showed significantly shorter stride length, *t*(29) = 4.35, adjusted *p* = 0.0006, dz. = 0.79, 95%CI [0.38, 1.21], and step length, *t*(29) = 4.82, adjusted *p* = 0.0002, dz. = 0.88, 95%CI [0.45, 1.31]. Cadence was significantly higher during DT2, *t*(29) = −4.96, adjusted *p* = 0.0001, dz. = −0.91, 95%CI [−1.34, −0.47]. Stance phase duration, *t*(29) = 4.46, adjusted *p* = 0.0004, dz. = 0.81, 95%CI [0.40, 1.23], and swing phase duration, *t*(29) = 4.21, adjusted *p* = 0.0008, dz. = 0.77, 95%CI [0.36, 1.18], were also significantly shorter during DT2 than during ST.

When DT2 was compared with DT1, further decreases were observed in stride length, *t*(29) = 2.41, adjusted *p* = 0.044, dz. = 0.44, 95%CI [0.06, 0.82], and step length, *t*(29) = 2.58, adjusted *p* = 0.032, dz. = 0.47, 95% CI [0.09, 0.85]. Cadence was significantly higher during DT2 than during DT1, *t*(29) = −2.69, adjusted *p* = 0.026, dz. = −0.49, 95%CI [−0.88, −0.11]. Significant reductions were also observed in stance phase duration, *t*(29) = 2.37, adjusted *p* = 0.047, dz. = 0.43, 95%CI [0.05, 0.81], and swing phase duration, *t*(29) = 2.49, adjusted *p* = 0.039, dz. = 0.45, 95%CI [0.07, 0.84].

Overall, the gait results showed progressively greater changes from ST to DT1 and DT2, particularly for stride length, step length, and cadence, whereas changes in stance and swing phase duration were not significant during DT1 relative to ST but became significant during DT2, as shown in Table 6.

**Table 6:** Walking gait parameters with different cognitive loads.

Gait parameters	ST	DT1	DT2
Stride length/cm	63.96 ± 5.06	59.77 ± 3.70*	57.54 ± 6.09***
Step length/cm	132.27 ± 8.90	124.65 ± 6.88*	118.17 ± 7.04***^#^
Cadence/(steps·min^−1^)	96.31 ± 6.41	102.00 ± 5.61*	107.69 ± 6.45***^#^
Stance phase/s	0.822 ± 0.039	0.807 ± 0.034	0.770 ± 0.042***^#^
Swing phase/s	0.296 ± 0.054	0.271 ± 0.052	0.229 ± 0.026***^#^

* indicates *p* < 0.05 compared with single-task walking; *** indicates *p* < 0.001 compared with single-task walking; # indicates *p* < 0.05 compared with 1-back walking; *n* = 30.

#### 3.2.2 Motor dual-task cost

Significant differences between DT1 and DT2 were observed in the DTCs of stride length, step length, cadence, and stance phase duration. Specifically, stride length DTC differed significantly between DT1 and DT2, *t*(29) = −2.74, adjusted *p* = 0.023, Cohen’s dz. = −0.50, 95%CI [−0.89, −0.11]. A significant difference was also observed for step length DTC, *t*(29) = −2.91, adjusted *p* = 0.023, dz. = −0.53, 95%CI [−0.92, −0.14]. Cadence DTC differed significantly between the two dual-task conditions, *t*(29) = 2.63, adjusted *p* = 0.023, dz. = 0.48, 95%CI [0.10, 0.87]. Similarly, a significant difference was found for stance phase DTC, *t*(29) = −2.49, adjusted *p* = 0.024, dz. = −0.45, 95%CI [−0.84, −0.07]. In contrast, no significant difference was observed in swing phase DTC between DT1 and DT2, *t*(29) = −1.46, adjusted *p* = 0.155, dz. = −0.27, 95%CI [−0.63, 0.10] (see Figure 6).

**Figure 6:**
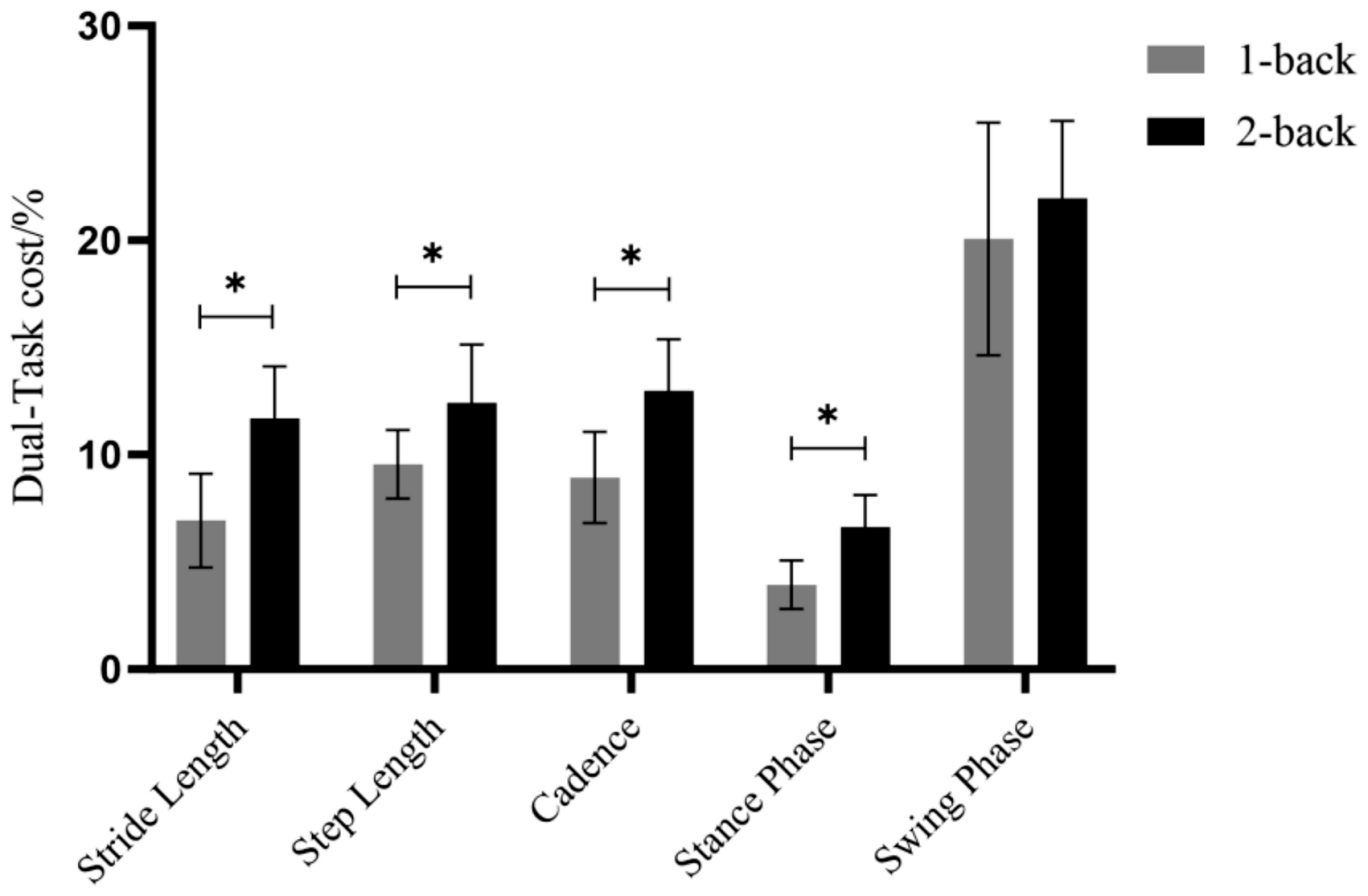
Motion dual-task cost. *p < 0.05.

#### 3.2.3 Cognitive performance

Table 7 shows auditory digit n-back task accuracy.

**Table 7:** Auditory digit n-back task accuracy.

Measure	1-back		2-back	
	CO1	DT1	CO2	DT2
Accuracy/%	99.6 ± 0.5	98.5 ± 3.4	97.3 ± 1.5	93.4 ± 5.1*^#^

* indicates *p* < 0.05 compared with single-task condition; # indicates *p* < 0.05 compared with 1-back walking; *n* = 30.

#### 3.2.4 Dual task costs

The cognitive dual-task cost was calculated based on accuracy. Cognitive dual-task cost was significantly greater during DT2 than during DT1, *t*(29) = 3.46, *p* = 0.0017, Cohen’s dz. = 0.63, 95%CI [0.24, 1.02], as shown in Figure 7.

**Figure 7:**
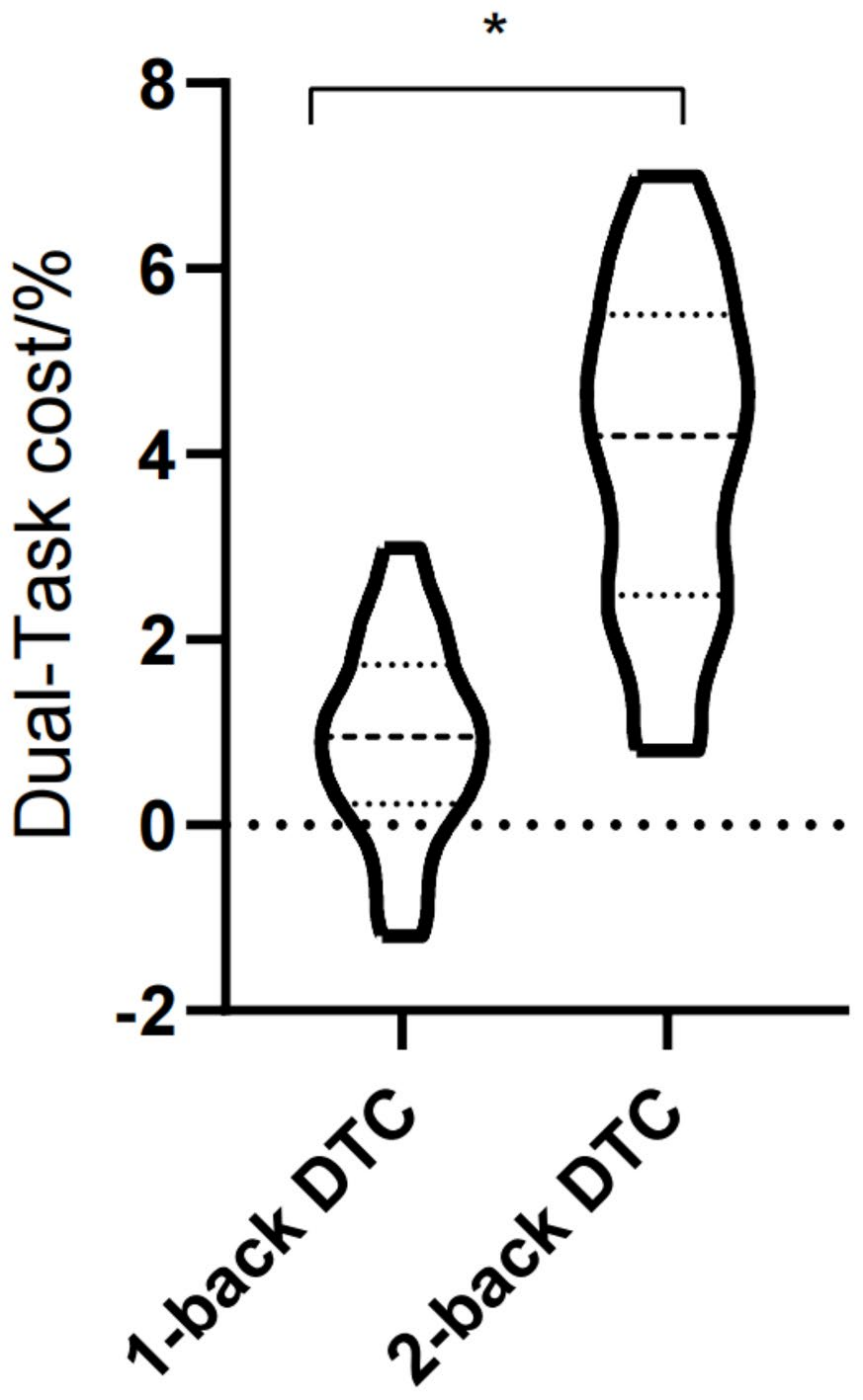
Cognitive dual-task cost. *p < 0.05.

For ease of evaluation, the orientation of the “task performance” dimension was ad- justed accordingly. Compared with ST, DT1 significantly increased mental demand (ST: 3.23 ± 2.05; DT1: 7.15 ± 5.23), effort (ST: 3.38 ± 1.85; DT1: 7.46 ± 5.11), and overall perceived workload (ST: 3.24 ± 1.57; DT1: 6.53 ± 2.12) (all *p* < 0.05). Furthermore, DT2 elicited the high est. workload levels, including mental demand (ST: 3.23 ± 2.05; DT1: 7.15 ± 5.23; DT2: 11.00 ± 4.28), physical demand (ST: 2.62 ± 1.12; DT1: 3.38 ± 1.76; DT2: 9.15 ± 3.41), temporal demand (ST: 3.23 ± 1.54; DT1: 5.62 ± 4.75; DT2: 10.46 ± 5.25), effort (ST: 3.38 ± 1.85; DT1: 7.46 ± 5.11; DT2: 11.46 ± 3.55), and frustration (ST: 2.08 ± 1.61; DT1: 3.38 ± 2.73; DT2: 6.62 ± 3.71), all of which were significantly higher under DT2 than under ST and DT1 (all *p* < 0.001). In contrast, perceived task performance scores progressively decreased across conditions (ST: 12.92 ± 1.61; DT1: 11.46 ± 2.18; DT2: 8.46 ± 2.67, *p* < 0.001), indicating poorer perceived performance with increasing cognitive load.

The overall perceived workload showed an increasing trend across the three conditions (ST: 3.24 ± 1.57; DT1: 6.53 ± 2.12; DT2: 9.53 ± 2.97, *p* < 0.001), as shown in Figure 8 and Table 8.

**Figure 8:**
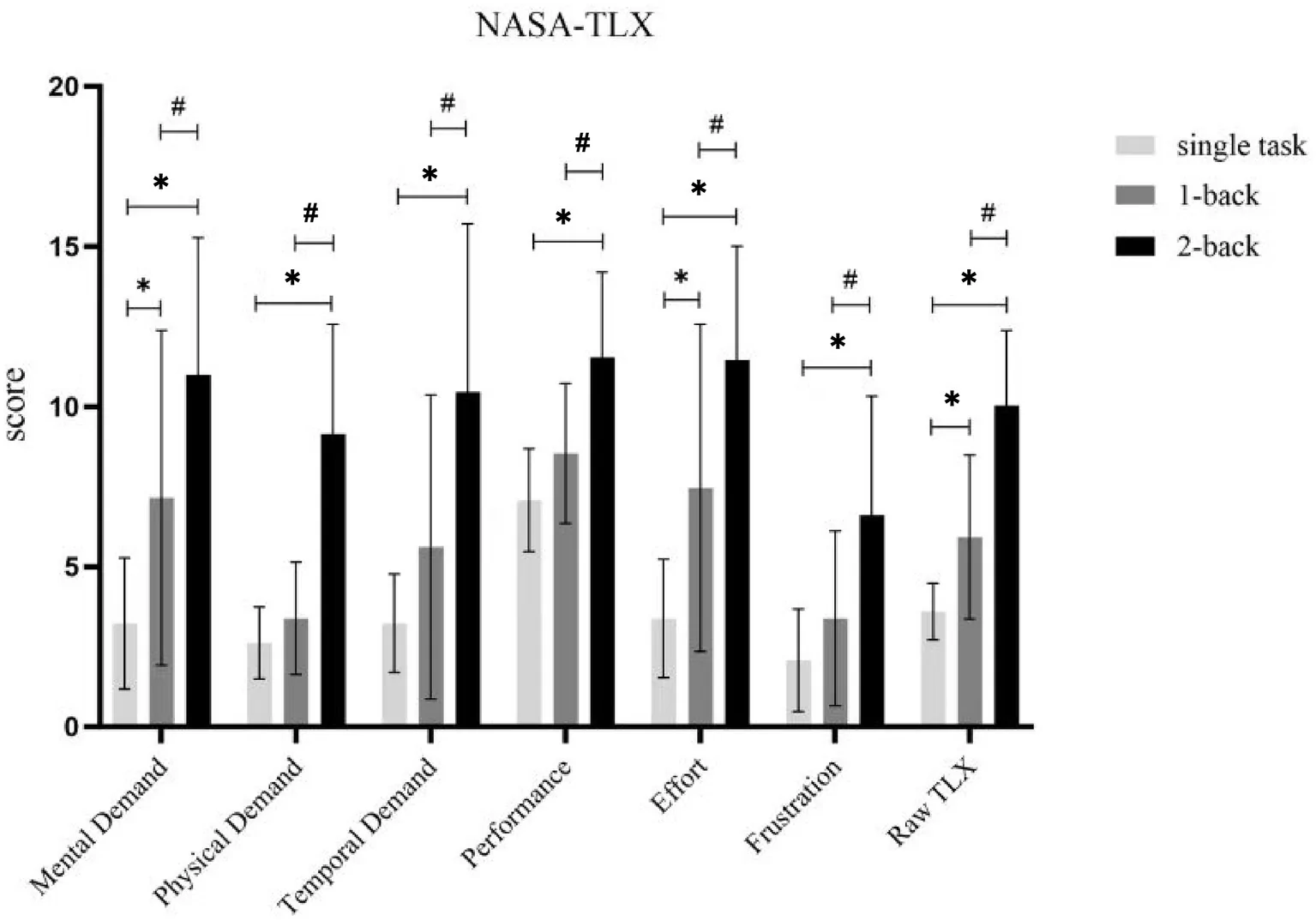
Perceived workload.

**Table 8:** Pairwise comparisons of NASA-TLX scores.

Dimension	Comparison	*t*	df	Adjusted *p*	Cohen’s dz	95%CI for dz
Mental demand	ST vs. DT1	3.12	29	0.006	0.57	[0.18, 0.96]
Mental demand	ST vs. DT2	7.26	29	<0.001	1.33	[0.82, 1.83]
Mental demand	DT1 vs. DT2	4.21	29	<0.001	0.77	[0.36, 1.18]
Effort	ST vs. DT1	3.34	29	0.004	0.61	[0.22, 1.00]
Effort	ST vs. DT2	8.02	29	<0.001	1.46	[0.92, 2.00]
Effort	DT1 vs. DT2	4.36	29	<0.001	0.80	[0.38, 1.21]
Perceived task performance	ST vs. DT2	−7.04	29	<0.001	−1.29	[−1.79, −0.79]
Perceived task performance	DT1 vs. DT2	−5.15	29	<0.001	−0.94	[−1.38, −0.50]
Overall perceived workload	ST vs. DT1	5.42	29	<0.001	0.99	[0.54, 1.44]
Overall perceived workload	DT1 vs. DT2	5.01	29	<0.001	0.91	[0.48, 1.35]

#### 3.2.5 Correlation analysis

Pearson correlation analysis revealed that the correlation patterns between significantly activated cortical regions in the brain and each dimension of the NASA-TLX exhibited marked differences under various task conditions (see Table 9 for details). Under the ST, only a few significant correlations were observed, primarily involving the PPC. Specifically, activation in the RPPC showed a positive correlation with frustration, while activation in the LPPC was positively correlated with task performance. Under the DT1, negative correlations predominated, mainly concentrated in the RM1, demonstrating significant negative correlations between brain activation and mental demand, time demand, effort, and overall workload. Under the DT2, there was a widespread positive correlation between brain activation and subjective workload, primarily involving the sensorimotor and parietal regions, with multiple dimensions (such as mental demand, physical burden, effort, and overall workload) showing significant positive correlations with cortical activation.

**Table 9:** Cortical-level correlation between brain activation and NASA-TLX scores across task conditions.

Task type	ROI	Dimensions	*r*	*n*	df	P_FDR
ST	RPPC	Frustration	0.420	30	28	0.0270
ST	LPPC	Task performance	0.450	30	28	0.0205
DT1	RM1	Mental demand	−0.480	30	28	0.0158
DT1	RM1	Temporal demand	−0.520	30	28	0.0104
DT1	RM1	Effort	−0.600	30	28	0.0033
DT1	RM1	Overall workload	−0.550	30	28	0.0069
DT2	LS1	Mental demand	0.460	30	28	0.0195
DT2	LS1	Physical demand	0.500	30	28	0.0127
DT2	LS1	Task performance	0.440	30	28	0.0217
DT2	LS1	Effort	0.490	30	28	0.0130
DT2	LS1	Overall workload	0.530	30	28	0.0097
DT2	LPPC	Mental demand	0.430	30	28	0.0256
DT2	RPPC	Effort	0.470	30	28	0.0163

Pearson correlation analysis also indicated that, under different task conditions, significantly activated channels in the brain exhibited differentiated correlation patterns with each dimension of the NASA-TLX (see Table 10 for details). Overall, channel activation tended to show positive or negative correlations with multiple subjective workload metrics, while mostly demonstrating negative correlations with task performance.

**Table 10:** Channel-level correlation between brain activation and NASA-TLX scores across task conditions.

Task type	Channel	Dimensions	*r*	*n*	df	P_FDR
ST	18	Mental demand	−0.520	28	26	0.0145
ST	18	Temporal demand	−0.500	28	26	0.0137
ST	18	Effort	−0.560	28	26	0.0105
ST	18	Overall workload	−0.610	28	26	0.0066
ST	30	Task performance	0.430	30	28	0.0306
DT1	33	Frustration	0.410	29	27	0.0332
DT1	43	Temporal demand	−0.430	27	25	0.0308
DT1	43	Overall workload	−0.450	27	25	0.0308
DT2	35	Mental demand	−0.460	27	25	0.0260
DT2	35	Temporal demand	−0.470	27	25	0.0200
DT2	35	Overall workload	−0.490	27	25	0.0122

Under the ST, the correlations were the most concentrated, primarily manifesting as significant negative correlations between Channel 18 and multiple subjective workload metrics. Additionally, Channel 30 showed a positive correlation trend with task performance. Under the DT1, only a few channels exhibited moderate correlation trends with subjective workload metrics. Under the DT2, brain-behavior correlations further differentiated, with some channels showing moderate negative correlation trends with subjective workload metrics. Overall, as task load increased, the correlation patterns at the channel level gradually shifted from concentrated and significant correlations to more dispersed and weakened correlations.

## 4 Discussion

### 4.1 Cortical activation under different cognitive loads

This study used fNIRS to investigate the effects of cognitive–motor dual-task walking under different cognitive load levels on cortical activation. FC, gait performance, and cognitive cost in healthy young adults. Compared with DT1, DT2 resulted in significantly higher cognitive and walking costs and poorer cognitive performance, indicating greater dual-task interference under higher cognitive load. Significant differences in cortical activation were observed across task conditions, particularly in the PFC, DLPFC, M1, and LPPC. In addition, FC analysis revealed task-dependent changes in connectivity strength among several cortical regions. Overall, increasing cognitive load altered both regional brain activation and interregional functional interactions during dual-task walking.

Compared with DT1, DT2 was characterized by reduced activation in the PFC and DLPFC despite higher cognitive demands. As key regions involved in executive control, attentional allocation, and working memory, the PFC is highly sensitive to changes in cognitive load during dual-task performance. Previous theoretical frameworks, including the Compensation-Related Utilization of Neural Circuits Hypothesis (CRUNCH) and inverted-U models of working-memory load, have been used to interpret similar load-dependent changes in prefrontal activation (Reuter-Lorenz and Cappell, 2008; Vijayraghavan et al., 2007; Callicott et al., 1999). From this perspective, the lower PFC and DLPFC activation observed during DT2 may reflect an altered pattern of prefrontal recruitment as cognitive demand increased, accompanied by higher cognitive dual-task cost and poorer cognitive performance.

Significant differences in HbO concentration were observed across the PFC, DLPFC, M1, and LPPC, indicating region-specific cortical responses to increasing cognitive load. While the PFC and DLPFC are mainly involved in executive control and working memory (Hamacher et al., 2015; Wang et al., 2023), M1 supports motor planning and execution (Ou et al., 2024; Ding et al., 2024; Rubenstein and Rakic, 2020). The sustained increase in M1 activation suggests greater recruitment of motor resources to maintain gait performance under increasing task demands, whereas activation changes in the LPPC may reflect enhanced sensorimotor integration. These findings indicate coordinated but functionally distinct contributions of prefrontal, motor, and parietal regions during cognitive–motor dual-task walking. Consistent with the ROI findings, channel level analyses also revealed significant activation changes in the PFC and M1. Increased activation in prefrontal channels may reflect greater executive demands (Degutis et al., 2024), whereas activation in motor channels may support gait performance under higher cognitive loads (Toriyama et al., 2026). Together, these findings suggest that increasing task difficulty modulates neural activity at both regional and channel-specific levels, supporting cognitive–motor integration during dual-task walking (Wang et al., 2020; Li et al., 2025).

### 4.2 Load-related functional connectivity changes during dual-task walking

The FC analysis revealed an overall increase in connectivity strength across task conditions, with FC being lowest during ST, increasing under DT1, and remaining elevated under DT2. This pattern is consistent with previous reports of task-dependent changes in interregional coupling (Fishburn et al., 2014). Specifically, the prefrontal–parietal network (e.g., RPFC–RPPC, RDLPFC–RPPC, and LPFC–LPPC) showed enhanced connectivity under dual-task conditions, indicating increased recruitment of executive-control and working-memory networks as cognitive load increased (Pongpipat et al., 2021; Yuk et al., 2020; Webler et al., 2022). In addition, significant enhancement was observed within the sensorimotor network, particularly in connections such as RM1–RS1 and LM1–RS1. These findings, together with increased activation of motor cortical regions, suggest greater involvement of sensorimotor integration during dual-task walking. Recent EEG studies of postural control indicate that balance regulation is accompanied by dynamic changes in directed cortical interactions among somatosensory, motor, parietal, and visual regions when sensory information becomes conflicting or unreliable (Wang et al., 2024, 2025a,b), which provide complementary evidence that postural control involves distributed cortical interactions relevant to sensory integration and balance regulation. Interestingly, most FC measures did not differ significantly between DT1 and DT2, although both were significantly higher than ST. This indicates that the transition from single to dual-task walking was accompanied by a marked increase in interregional connectivity, whereas increasing working-memory load from 1-back to 2-back produced relatively limited additional changes. Recent postural-control studies similarly show that cortical network interactions vary according to sensory and balance demands rather than changing uniformly with task difficulty (Wang et al., 2024, 2025a,b). Taken together, these findings suggest that interregional functional connectivity was primarily altered by the transition from single- to dual-task walking, with relatively limited additional changes as working-memory load increased further. Although PFC activation decreased under DT2, FC remained elevated, further indicating that regional activation and interregional connectivity may show different load-related patterns.

### 4.3 Task performance and neural correlates of cognitive load

As task difficulty increased, enhanced FC was accompanied by deteriorated cognitive and motor performance, indicating that stronger interregional functional connectivity occurred alongside greater dual-task interference. The posture-first strategy and attentional resource theories provide useful frameworks for interpreting these concurrent neural and behavioral changes (Crowell et al., 2020; Yogev‐Seligmann et al., 2012; Shumway-Cook et al., 1997). In the present study, the narrow-base walking condition increased balance-control demands, requiring additional sensory, cognitive, and neuromuscular resources while participants simultaneously performed the n-back task (Patelaki et al., 2023; Ruffieux et al., 2015). In the present study, the narrow-base walking condition increased balance-control demands, requiring additional sensory, cognitive, and neuromuscular resources while participants simultaneously performed the n-back task (Hennah et al., 2021). Consistent with this increased task demand, DT2 was characterized by higher perceived workload, lower cognitive accuracy, and greater deterioration in motor performance (Hoang et al., 2020; Rahman et al., 2021; Fraser et al., 2016). These findings suggest that increasing working-memory demands intensified competition for limited processing resources, while interregional functional connectivity remained elevated. This pattern is consistent with capacity-limitation and resource-competition accounts of dual-task performance (Schaefer, 2014; Kelly et al., 2012; Wu and Wang, 2024), and highlights the coexistence of stronger functional connectivity and declining behavioral performance under higher cognitive load.

Pearson correlation analysis revealed task-dependent relationships between cortical activation and subjective cognitive load. As task complexity increased, the correlation pattern differed across ST, DT1, and DT2, indicating that the association between cortical activation and perceived workload varied with task demands (Xu et al., 2024). Previous studies have similarly shown that changes in cortical activation and subjective workload during walking are sensitive to variations in cognitive and dual-task demands (Hoang et al., 2020; Lin and Lin, 2016). Under ST, only a limited number of significant associations were identified. Under DT1, activation in RM1 was negatively associated with several workload dimensions, whereas under DT2, positive associations were observed mainly in sensorimotor and parietal regions. These task-dependent correlation patterns suggest a load-related shift in the cortical regions associated with perceived workload, with increasing cognitive demand involving broader sensorimotor and parietal contributions to workload-related processing (Lim et al., 2022; Pizzamiglio et al., 2017).

Methodologically, the present study extends previous cognitive–motor dual-task research by parametrically manipulating working-memory load within the same auditory n-back paradigm while keeping the walking task constant. In addition, cortical activation, ROI-to-ROI functional connectivity, gait and cognitive performance, dual-task costs, and subjective workload were evaluated within the same experimental framework. This integrated design provides a multidimensional characterization of load-related neural and behavioral changes during dual-task walking.

### 4.4 Limitations and future directions

Several limitations constrain the interpretation of the present findings. First, the sample consisted of 30 healthy college students aged 18–25 years and did not include older adults or clinical populations. Because age and clinical status can influence cortical activation, functional connectivity, gait control, and cognitive-motor interference, the findings should not be generalized beyond healthy young adults without direct replication. The relatively small sample size may also limit statistical power, particularly for moderate effects and brain-behavior correlations. Second, this study employed n-back tasks (*n* = 1, 2) to manipulate cognitive load in a graded manner. However, using only two load levels may be insufficient to comprehensively characterize the nonlinear relationship between cognitive load and motor performance. For instance, whether brain activation patterns and gait regulation exhibit plateau effects or inflection points under higher load conditions (e.g., *n* ≥ 3) remains to be further verified. In addition, although the present study examined both global mean functional connectivity and specific ROI-to-ROI connections, node-specific graph-theoretical metrics and seed-based functional connectivity analyses were not included. Previous fNIRS studies have shown that node or seed-specific connectivity measures may provide additional information regarding the contribution of individual cortical regions and specific connectivity pathways (Yang et al., 2020; Ji et al., 2020). Future studies incorporating measures such as node degree, local efficiency, and seed-based connectivity may provide a more detailed characterization of load-related changes in cortical network organization during cognitive-motor dual-task walking. Finally, although fNIRS technology offers advantages such as portability and suitability for dynamic tasks, its spatial resolution is relatively limited. Moreover, head movements during dynamic experimental conditions, such as walking, may introduce a degree of motion artifacts. In addition, fNIRS measurements may be influenced by extracerebral and systemic physiological fluctuations, as noted in previous methodological work (Ferrari and Quaresima, 2012). Despite the application of signal filtering and motion correction methods in this study, the potential impact of residual noise on the accuracy of blood oxygen signal estimation cannot be entirely ruled out.

## 5 Conclusion

In healthy young adults, increasing working-memory demands during narrow-base walking were associated with greater cognitive and walking dual-task costs, region-specific changes in cortical activation, and stronger functional connectivity in several prefrontal-parietal and sensorimotor connections. These findings suggest that cortical activation, functional connectivity, and behavioral performance may show distinct load-related patterns during cognitive-motor dual-task walking. A key contribution of this study is the integrated assessment of graded working-memory load, cortical activation, multi-region functional connectivity, dual-task costs, and subjective workload while keeping the walking task constant. This framework provides a multidimensional characterization of load-related neural and behavioral changes and may inform the design of more precisely graded dual-task paradigms. Future studies should extend these findings across broader cognitive load ranges and aging or clinical populations.

## Data Availability

The raw data supporting the conclusions of this article will be made available by the authors, without undue reservation.
